# Complex Permittivity Characterization of Liquid Samples Based on a Split Ring Resonator (SRR)

**DOI:** 10.3390/s21103385

**Published:** 2021-05-12

**Authors:** Jialu Ma, Jingchao Tang, Kaicheng Wang, Lianghao Guo, Yubin Gong, Shaomeng Wang

**Affiliations:** School of Electronic Science and Engineering, University of Electronic Science and Technology of China, Chengdu 610054, China; littlehorses0592@gmail.com (J.M.); tangjcjc@outlook.com (J.T.); kaicheng_wang@std.uestc.edu.cn (K.W.); 201911022440@std.uestc.edu.cn (L.G.); ybgong@uestc.edu.cn (Y.G.)

**Keywords:** microwave sensor, liquid sample, SRR, complex permittivity characterization

## Abstract

A complex permittivity characterization method for liquid samples has been proposed. The measurement is carried out based on a self-designed microwave sensor with a split ring resonator (SRR), the unload resonant frequency of which is 5.05 GHz. The liquid samples in capillary are placed in the resonant zone of the fabricated senor for high sensitivity measurement. The frequency shift of 58.7 MHz is achieved when the capillary is filled with ethanol, corresponding a sensitivity of 97.46 MHz/μL. The complex permittivity of methanol, ethanol, isopropanol (IPA) and deionized water at the resonant frequency are measured and calibrated by the first order Debye model. Then, the complex permittivity of different concentrations of aqueous solutions of these materials are measured by using the calibrated sensor system. The results show that the proposed sensor has high sensitivity and accuracy in measuring the complex permittivity of liquid samples with volumes as small as 0.13 μL. It provides a useful reference for the complex permittivity characterization of small amount of liquid chemical samples. In addition, the characterization of an important biological sample (inositol) is carried out by using the proposed sensor.

## 1. Introduction

Over the past decades, material characterization in the microwave/RF range, owing to the great potential application in many areas such as biomedical, chemical, food safety and agriculture fields [1,2,3,4] has been attracting more and more interest. Nowadays, complex permittivity measurements of liquid samples play an important role in various biomedical and chemical applications [5,6]. Researchers have developed many methods for characterizing the complex permittivity of certain samples, such as free space methods, transmission/reflection methods and the resonant methods, etc., [7,8,9].

The non-resonant methods such as free space methods and transmission/reflection methods provide performing measurements for a great amount of material in large bandwidth, but the measurement accuracy and sensitivity are not precise enough. While the resonant methods based on the perturbation of the introduced sample to the electric field distribution of the resonant structure to achieve the sensing purpose [10], thus, the resonant methods can provide higher accuracy with smaller samples than the non-resonant methods. Because the sensor area is in the concentrated electromagnetic field, which means the material under test (MUT) can cause larger perturbation of the electromagnetic field. Meanwhile, the permittivity characterization methods based on resonance structure provide a quite convenient measurement progress of the resonant frequency, peak attenuation and quality factor which can be directly read out from the S-parameters. Therefore, researchers have proposed a variety of resonant structures for the characterization of complex permittivity, such as cavity device [11,12] and substrate integrated waveguide (SIW) device [13,14]. A rectangular cavity device based on improved cavity perturbation method at the range of 26.5–40 GHz has been proposed in our previous work [15]. In the field of biological and chemical, the characterization of small volume liquid samples has received more and more attentions. Many characterization methods have been proposed, some of which are based on complex micro-channels and resonant structures, leading to cost increase and complication of the measurement process. Then the microfluidic technology becomes popular as it provides an effective path for the characterization of small volume liquid samples [16,17,18]

In recent years, the planar split ring resonator (SRR) has attracted attentions for its high sensitivity from the microwave/RF [19,20,21] to the THz range [22,23,24]. Furtherly, different structures based on SRR have been proposed by many researchers. Chretiennot et al. [25] presented a novel planar resonance structure based on microfluidic technology to achieve the sensing of liquid samples. Ali et al. [26] proposed a high Q sensor based on SRR with sparkline filters for the solid materials characterization. Lee et al. [27] reported a single element planar SRR based biosensor for DNA hybridization detection at microwave regime. Generally, plane microwave sensors based on SRR can exhibit a strong localization and enhancement of fields to improve the sensor sensitivity and reduce the amount of samples. Therefore, microwave sensor based on SRR have been widely used in the sensing field for the advantage of lower cost, less samples, and higher sensitivity. In this paper, an improved microwave sensor based on planar SRR with a resonant frequency of 5.05 GHz is proposed and fabricated, its sensitivity for small volume liquid samples has been investigated. The sensor system is label free, real time and non-destructive. In addition, the modules in the measurement system are reusable and easily fabricated, which can effectively reduce the cost of the measurement.

The manuscript is organized as follows: In Section 2, the proposed microwave sensor based on resonant perturbation method for the material characterization is discussed in details. In Section 3, the permittivity characterization method for liquid samples is introduced. Section 4 and Section 5 present the experiment validation of the proposed sensor. A conclusion is given at the end.

## 2. Materials and Methods

### 2.1. Sensor Design

**Figure 1:**
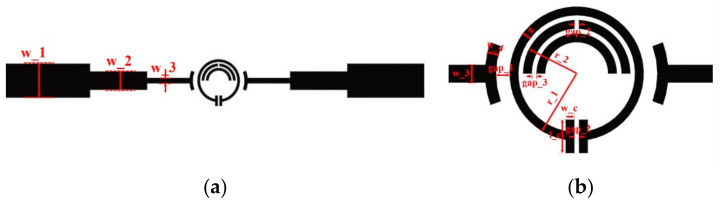
Structure of the proposed microwave sensor. (**a**) The full view of the microwave sensor; (**b**) the SRR structure and the sensing zone of the sensor.

A three-stage coupled two-port SRR structure for the characterization of liquid sample is proposed, as shown in Figure 1a,b.

**Figure 2:**
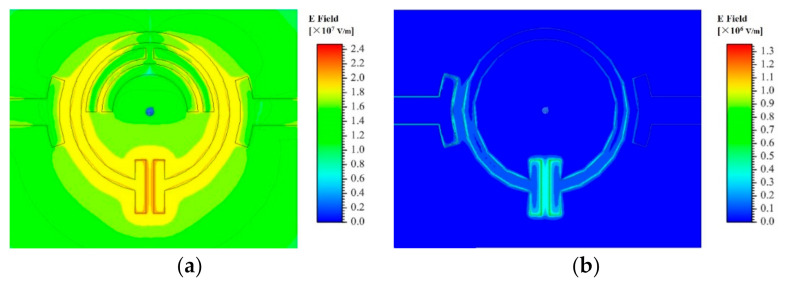
Electric distribution of SRR structure (**a**) with inner metal rings and (**b**) without inner metal rings.

The SRR consists of three metal rings. The outer metal ring is a circular structure with an opening gap at one side. At the same time, two parallel rectangular structure form the sensing area at the opening side; the inner metal ring is two semicircular structures, one of which has the opening gap at the top of the ring. The electric field distributions of the SRR with and without inner metal rings which is simulated by using the High Frequency Structure Simulator (HFSS) software as shown in Figure 2. As can be seen, compared to the single-ring structure, the proposed three-ring structure show a denser electric field concentration at the rectangular sensing area, which means a higher quality factor and a better sensitivity.

The three-stage coupling microstrip line is designed based on the Chebyshev coupling transformation [28] for the purpose of impedance transformation. The impedance of the coupling line is gradually changed from the standard 50 Ohm to the impedance required for the optimal coupling with the proposed SRR, which can guarantee the quality factor of the microwave sensor is maximized and provide the highest sensitivity. One end of the coupling microstrip line is connected to the standard SMA connector with a coupling impedance of 50 Ohm, and the other end is connected to the SRR structure through slot coupling. The coupling strength of the microstrip line and the SRR structure is related to the distance between them, and the coupling strength directly affects the quality factor of the sensor. Therefore, in order to improve the sensitivity of the sensor, the distance between the coupling microstrip line and the SRR structure should be optimized to obtain the required level of coupling.

**Figure 3:**
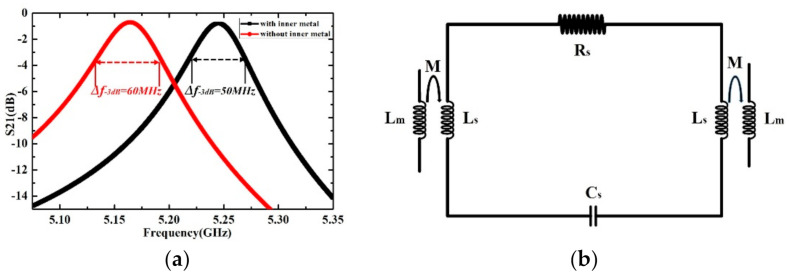
(**a**) Numerically resolved transmission magnitude curve of the microwave sensor (**b**) Equivalent circuit with Lm for the inductance of the microstrip coupling line, RLC for the parasitic elements of the SRR structure, and M for the mutual inductance between the coupling line and the SRR structure.

The design work is then carried out by using the High Frequency Structure Simulator (HFSS) software and all the structural parameters of the sensor are listed in Table 1. The electromagnetic wave of the quasi-TEM mode is coupled from the three-stage coupling line to the SRR structure and then coupled out from the other side. The resonance occurs when the electric energy stored in the SRR gap is equal to the magnetic energy in the SRR loop. The Numerically resolved transmission magnitude curve of the microwave sensor with and without the inner metal rings are shown in Figure 3a. The sensor with inner metal rings shows a narrower −3 dB bandwidth, which means the higher Q factor of the sensor. The Q factors of the SRR structure with and without inner metal rings are 104.2 and 86.0, respectively.

As shown in Figure 2a, a strong electric field distribution is established in the rectangular sensing area. The resonance can also be observed from the transmission response curve in Figure 3a. In quasi-static approximation, the sensor system composed of a SRR structure and two transmission lines can be approximated by basic RLC circuit elements, as shown in Figure 3b. The impedance of the resonator can be described as:

Z_s_ = R_s_ + jωL_s_ + 1/jωC_s_ (1)

where R_s_, L_s_ and C_s_ represent the impedance, inductance and capacitance of the SRR, respectively. The capacitance C_s_ can be easily affected by the complex permittivity of the liquid sample around the rectangular sensing area, and can be roughly approximated by:

C_s_ = C_0_ + ***ε***_sam_C_1_ (2)

where C_0_ represents the capacitive effect from the substrate and surrounding space. The term ***ε***_sam_C_1_ represents the contribution from the loaded sample with C_1_ for the capacitance of the empty capillary channel and ***ε***_sam_ = ***ε***′_sam_ + j***ε***″_sam_ for the complex permittivity of liquid sample. Then the total resistance R_all_ and total capacitance C_all_ of the all SRR structure and capillary channel are functions of the complex permittivity of the liquid sample, and the resonance frequency of the sensor system can be represented as:

(3) $$f_{0}=1/2\pi \sqrt{L_{s}C_{\mathrm{all}}}$$

(4) $$Q=\sqrt{L_{s}/C_{\mathrm{all}}}/R_{\mathrm{all}}$$

From Equations (3) and (4), it is clear that the microwave sensor resonance is dominated by the complex permittivity of the sample under test. By analyzing the correlation, the complex permittivity of sample can be calculated from the measurable transmission curve.

**Figure 4:**
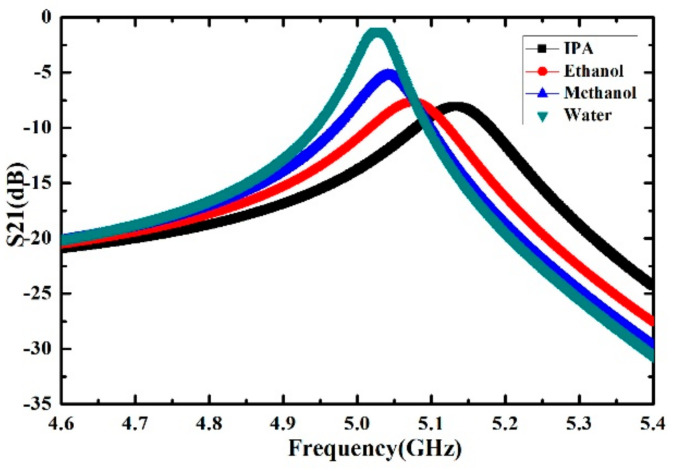
Simulated transmission magnitude curve of the microwave sensor with different liquid samples (IPA, ethanol, methanol and deionized water).

The simulation results of the proposed microwave sensor with different liquid samples (IPA, ethanol, methanol and deionized water) located on the rectangular sensing area are shown in Figure 4. The complex permittivities of the liquid samples are obtained by Debye model at the resonant frequency from [29,30]. The results show that liquid samples with different complex permittivities correspond to different frequency shift and Q factor. Therefore, the quantitative calculation of complex permittivities for different liquid samples can be achieved by using the proposed microwave sensor.

### 2.2. Fabrication and Measurement

In order to verify the performance of the proposed microwave sensor for the characterization of liquid samples, and to illustrate the relationship between the resonance frequency and the complex permittivity of the liquid samples, we fabricated the microwave sensor using the standard chemical etching techniques.

**Figure 5:**
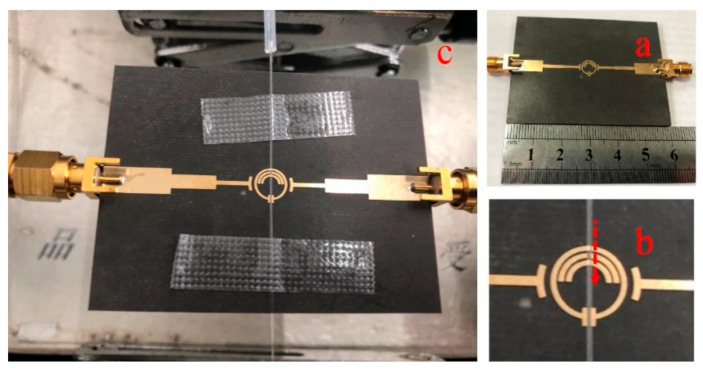
Assembled sensor system. (**a**) Fabricated microstrip coupled SRR with two SMA connectors; (**b**) Zoom-in view of the capillary channel attached to the sensing area. The channel is laid onto the SRR structure and the arrow indicates the flow direction; (**c**) Fully view of the assembled sensor with quartz tubes delivering liquid in the capillary channel and coaxial cables connected to the VNA.

The fabricated structure of the microwave sensor is shown in Figure 5. Rogers/Duriod 5880 with the thickness of 1.57 mm, dielectric constant of 2.2 and loss tangent of 0.0009 is chosen as the substrate. Rogers/Duriod 5880 is widely used in MW/RF circuit design for the uniform electrical properties over wide frequency range and low electrical loss [31,32]. On the upper surface of the substrate, a 35 μm thick gold-coated copper film with an electrical conductivity of 5.71 × 10^7^ S/m is deposited to construct the pattern of the microwave sensor. On the backside of the substrate, a 70 μm thick copper layer is deposited as the ground. A quartz capillary with inner diameter of 0.288 mm and outer diameter of 0.384 mm is used as the channel of the liquid sample. As shown in Figure 5c, the channel is manually positioned across the SRR structure gap so that the liquid sample can flow straight through the sensing area where the electric field is the strongest. The capillary channel made by quartz can ensure that the volume and shape of the liquid samples remain consistent during the measurement progresses. The resonance peak of the microwave sensor is directly affected by the relative position of the channel and the sensing area. Therefore, during the measurement progress, the position of the channel in the sensing area should remain unchanged to ensure an effective measurement.

**Figure 6:**
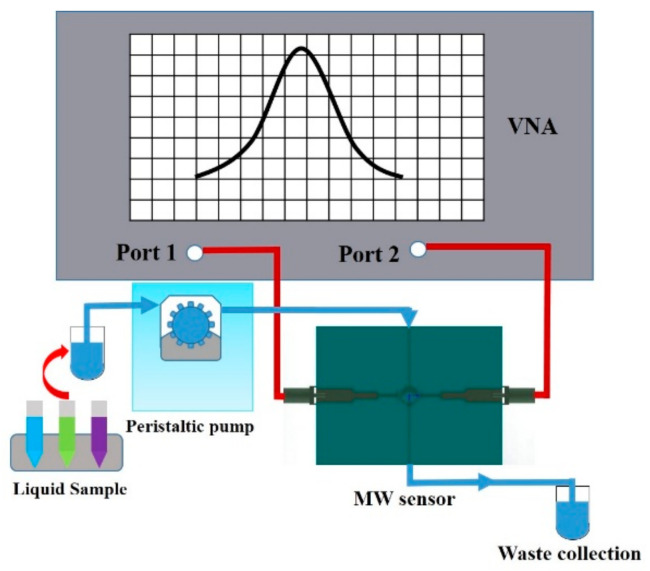
Schematic diagram of the experimental setup. The system is mainly composed of the VNA, peristaltic pump, waste collection and microwave sensor module.

The experimental setup is shown in Figure 6. The calibrated E8363B Vector Network Analyzer (Agilent, Santa Clara, CA, USA) is set to transmit measurement mode (S21) for the real time monitoring of the sensor response. At the same time, in order to achieve accurate measurement in the frequency range of 4.85–5.10 GHz, 6401 scanning points were selected. The stop-flow technique is employed in the measurement, which means the liquid sample were injected into the capillary channel by the peristaltic pump to keep the flow rate as a constant during all the measurement progress. The concentrated electric field in the rectangle sensing area ensure that any change of the liquid sample complex permittivity can be reflected by the change of the transmission resonance, and then recorded by the VNA.

**Figure 7:**
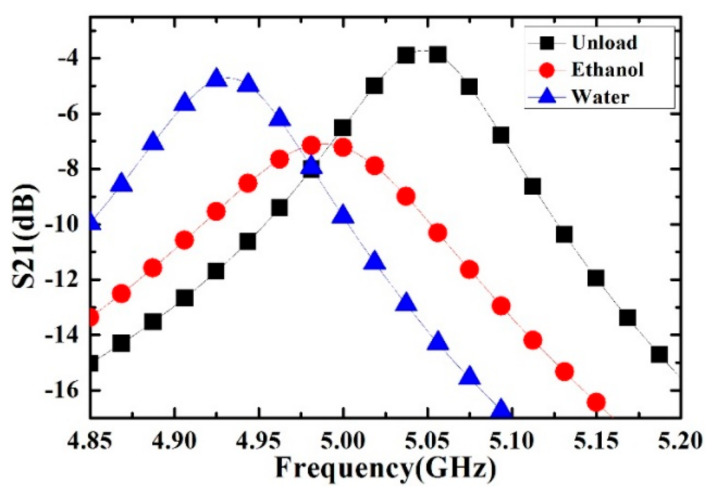
Measured transmission response of the microwave sensor. The curves represent different measurement conditions: the sensor with empty channel (unload), the sensor with channel filled with 100% ethanol, and 100% deionized water.

It has been experimentally verified that the continuous flow does not affect the measurement results, as long as the pressure is low enough to avoid the channel deformation. Each kind of liquid sample is measured for five times to reduce the measurement error. During the entire measurement process, the temperature and the humidity ware maintained at 25 °C and 40%, respectively. When the microwave SRR sensor is completely exposed to free space without a capillary channel above the gap, the resonance frequency and Q factor are the highest. When the capillary channel covers the electric field sensitive area of the gap, the resonance frequency and Q factor are slightly reduced. Depending on the complex permittivity of different liquid samples, the resonance characteristics of the microwave sensor change accordingly. The transmission responses of the microwave sensor are shown in Figure 7.

The black, red and blue dotted lines represent the resonance characteristics of capillary channel with air (unload), ethanol and deionized water, respectively. It can be seen from the results that a small volume of different kinds of liquid sample can cause observable changes in the resonance frequency and Q value.

## 3. Sensor’s Characteristic

The resonance frequency and Q factor of the microwave sensor are related to the complex permittivity of the liquid sample. The relationship can be described by a nonlinear function. It is difficult to accurately describe the functional relationship because of the complexity of the SRR structure and sensing area. Thus, we simplify the solving process by using a set of linear function to approximate the relationship. In this case, the simplified model related to the change of the complex permittivity and the sensor resonant frequency and Q factor can be expressed as:

(5) $$\left[\begin{array}{c} \Delta f\\ \Delta Q \end{array}\right]=\left[\begin{array}{cc} m_{11} & m_{12}\\ m_{21} & m_{22} \end{array}\right]\left[\begin{array}{c} \Delta \varepsilon \prime \\ \Delta \varepsilon \prime \prime \end{array}\right]$$

The parameters Δ*f* = *f*_sam_ − *f*_ref_ and ΔQ = Q_sam_ − Q_ref_ represent the change of resonant frequency and Q factor of the microwave sensor, respectively. The parameters Δ***ε***′ = ***ε***′_sam_ − ***ε***′_ref_ and Δ***ε***″ = ***ε***″_sam_ − ***ε***″_ref_ represent the real part and imaginary part of the liquid sample’s complex permittivity. The subscript ‘sam’ is for liquid sample and ‘ref’ is for reference sample. The parameters *m*_11_, *m*_12_, *m*_21_ and *m*_22_ is related to the electrical characteristics of the fabricated sensor. In the Equation (5), the relationship between resonance characteristics of the sensor and the complex permittivity of the liquid sample under test is described. Once we know the electrical parameters of the sensor, i.e., the values of parameters *m*_11_, *m*_12_, *m*_21_ and *m*_22_ in the Equation (5), the complex permittivity of the liquid sample under test can be calculated by the frequency shift and the Q-factor change.

### Calibration of the Sensor

In order to accurately characterize the complex permittivity of the liquid sample, the proposed microwave sensor should be first calibrated by using the liquids with known complex permittivity. In our paper, the ethanol, methanol, isopropanol and deionized water are used for the calibration. In each measurement step, the capillary channel is first filled with the liquid sample by using the stop-flow technique, and then the corresponding transmission response curve of one sample was recorded by VNA; then, the capillary channel is evacuated by nitrogen for the next sample measurement.

**Figure 8:**
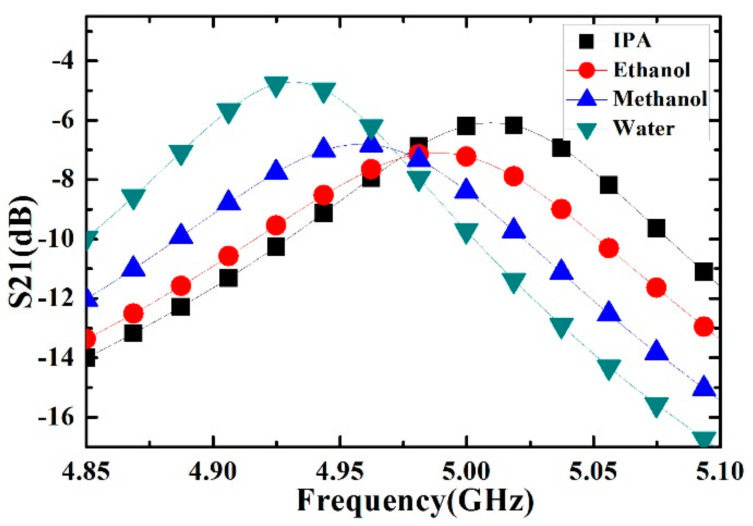
Measured transmission response of different liquid sample (isopropanol (IPA), ethanol, methanol and deionized water).

The measured transmission curves of the sensor calibrated by ethanol, methanol, isopropanol and deionized water are presented in the Figure 8. The measurement results show that the liquid samples such as IPA, ethanol, methanol and deionized water correspond to different frequency shifts and Q factors, which are quite consistent with the simulation results shown in Figure 4. The corresponding complex permittivity of liquid sample used for the calibration can be described by the first order Debye model as:

(6) $$\varepsilon_{\mathrm{sam}}=\varepsilon_{\infty }+\frac{\Delta \varepsilon_{D}}{1-j\omega \tau_{D}}$$

The high frequency relative permittivity ***ε***_∞_, dielectric decrement Δ***ε****_D_*_,_ and Debye relaxation time *τ_D_* of ethanol, methanol, isopropanol and deionized water are available in [29,30], just as shown in Table 2. Then the real and imaginary part of ethanol, methanol, isopropanol and deionized water can be calculated.

When we consider the calibration situation, the ***ε***_liqud_ represents the complex permittivity of ethanol, methanol, isopropanol and deionized water which can be calculated by Equation (6) and the parameter values in Table 2. The frequency shifts and Q-factor measured by the microwave sensor are listed in Table 3. According to Equation (5), a set of linear functions used for described the relationship of resonance characteristics of the sensor and the complex permittivity of the liquid sample under test can be accurately stated as follows:

(7) $$\left[\begin{array}{c} \Delta f\\ \Delta Q \end{array}\right]=\left[\begin{array}{cc} 1.8471 & -15.5735\\ -1.0702 & -5.0037 \end{array}\right]\left[\begin{array}{c} \Delta \varepsilon \prime_{\mathrm{sam}}\\ \Delta \varepsilon \prime \prime_{\mathrm{sam}} \end{array}\right]$$

In Equation (7), Δ***ε***′_sam_ = ***ε***′_liqud_ − ***ε***′_air_, Δ***ε***″_sam_ = ***ε***″_liqud_ − ***ε***″_air_, and ***ε***_liqud_ = ***ε***′_liqud_ + *j**ε***″_liqud_. By inverting the matrix in Equation (7), a mathematical model for determining the complex permittivity of unknown liquid sample is derived as:

(8) $$\left[\begin{array}{c} \Delta \varepsilon \prime_{\mathrm{sam}}\\ \Delta \varepsilon \prime \prime_{\mathrm{sam}} \end{array}\right]=\left[\begin{array}{cc} 0.6741 & -2.0982\\ -0.1442 & 0.2489 \end{array}\right]\left[\begin{array}{c} \Delta f\\ \Delta Q \end{array}\right]$$

By obtaining Δ***ε***′_sam_ and Δ***ε***″_sam_ from each pairs of measured resonance frequency shift and Q factor change, the complex permittivity of each sample is calculated as:

(9) $$\varepsilon \prime_{\mathrm{sam}}=\varepsilon \prime_{\mathrm{air}}+\Delta \varepsilon \prime_{\mathrm{sam}}$$

(10) $$\varepsilon \prime \prime_{\mathrm{sam}}=\varepsilon \prime \prime_{\mathrm{air}}+\Delta \varepsilon \prime \prime_{\mathrm{sam}}$$

Equations (8)–(10) can be used for the calculation of the complex permittivity of unknown liquid sample under test.

## 4. Complex Permittivity Characterization of Ethanol-Water Solution, Methanol-Water Solution and IPA-Water Solution

The complex permittivity of ethanol aqueous solution, methanol aqueous solution and IPA aqueous solution are calculated by the calibrated sensor in this section. The density and viscosity of ethanol, methanol, IPA and deionized water are relatively close, so the influence of density and viscosity on the resonant frequency and Q factor has been ignored in our experiment. The resonant characteristics of the microwave sensor with empty capillary channel is used as reference. Coupled thermometers and humidity were used to make sure no any change in the temperature and humidity during the whole measurement process.

**Figure 9:**
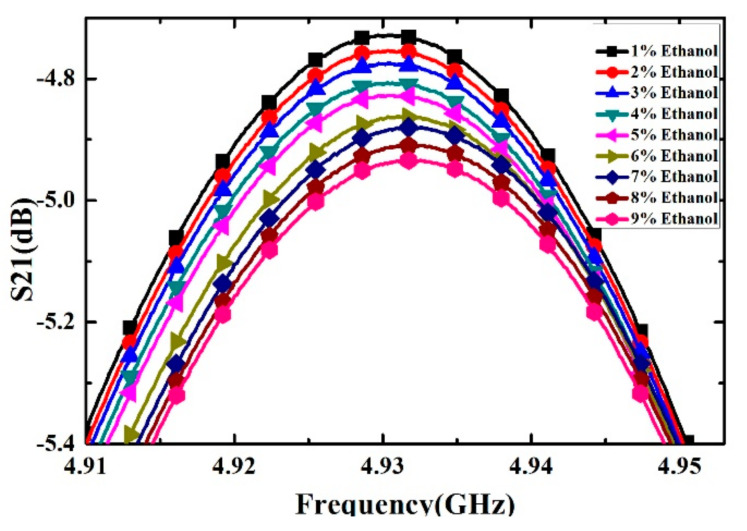
Measured transmission response of different concentrations of ethanol solution (from 1% to 9% with step of 1%).

**Figure 10:**
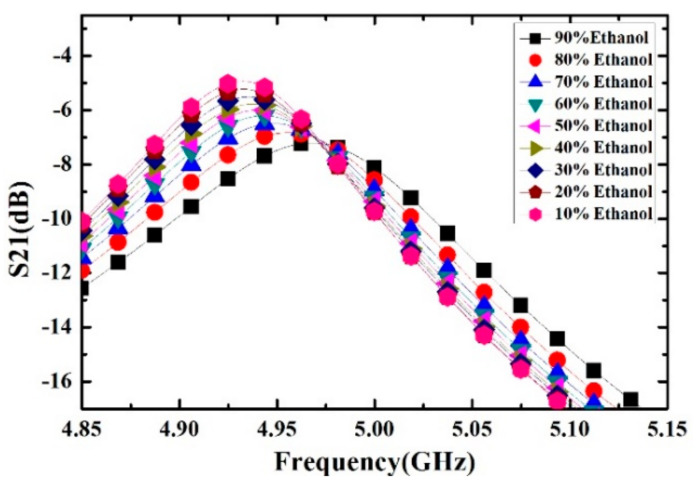
Measured transmission response of different concentrations of ethanol solution.

**Figure 11:**
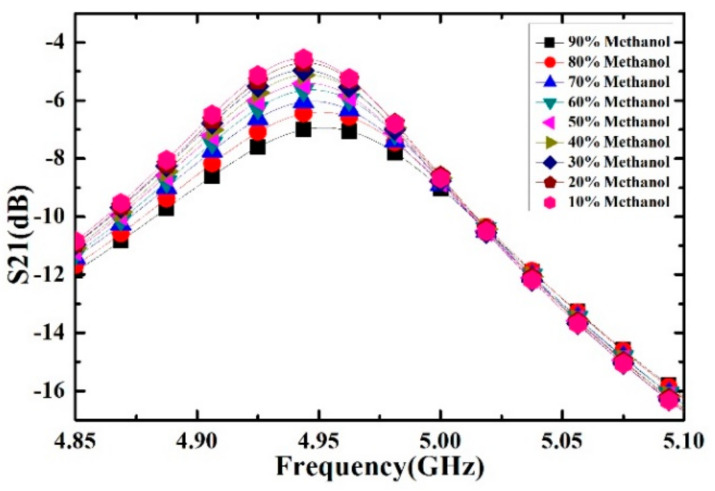
Measured transmission response of different concentrations of methanol solution.

**Figure 12:**
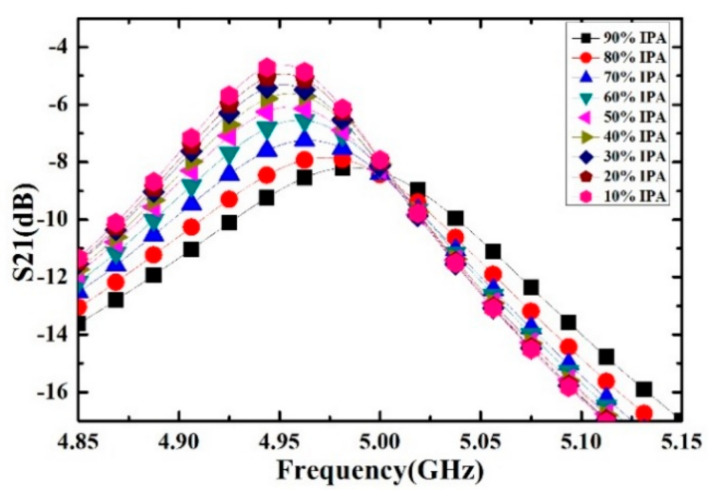
Measured transmission response of different concentrations of IPA solution.

Ethanol solutions with volume fractions from 1% to 9% with the step of 1% have been prepared and measured. For each volume fraction, the measurement has been carried out for 5 times to verify the repeatability and the average value is supposed to be the real result. The measured transmission response curves are given in Figure 9, from which the frequency shifts and Q factors are calculated and listed in Table 4. Ethanol solution, methanol solution and IPA solution with different volume fractions are prepared, which varied from 10% to 90% with the step of 10%. For the different concentrations of sample solution, the measured transmission response curves of the microwave sensor are given in Figure 10, Figure 11 and Figure 12, the frequency shift and the Q factor are listed in Table 5, Table 6 and Table 7. The results show that, for the sample, the transmission response curve shows a regular change with the increase of ethanol content.

**Figure 13:**
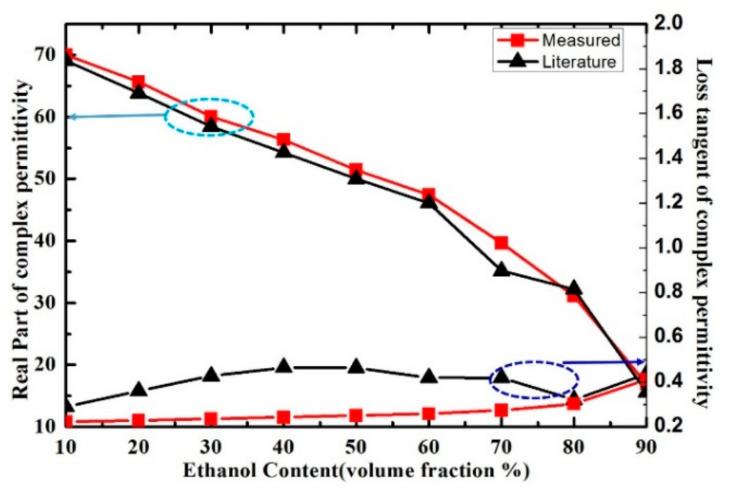
Measured and literature results of real part and loss tangent of ethanol-water mixture solution at the frequency of 5.05 GHz.

Then Equations (8)–(10) were used for the calculation of complex permittivity of the mixture ethanol-water, methanol-water and IPA-water solution from the parameter value listed in Table 5, Table 6 and Table 7. In the measurement, the ***ε***_sam_ represents the complex permittivity of different volume fraction of ethanol solution. The real part and loss tangent of different volume fractions of ethanol solutions calculated by Equations (8)–(10) are shown in Figure 13. The results show that, as the increase of ethanol content, the real part of the complex permittivity of ethanol-water solution decrease, while the loss tangent of the complex permittivity increase.

**Figure 14:**
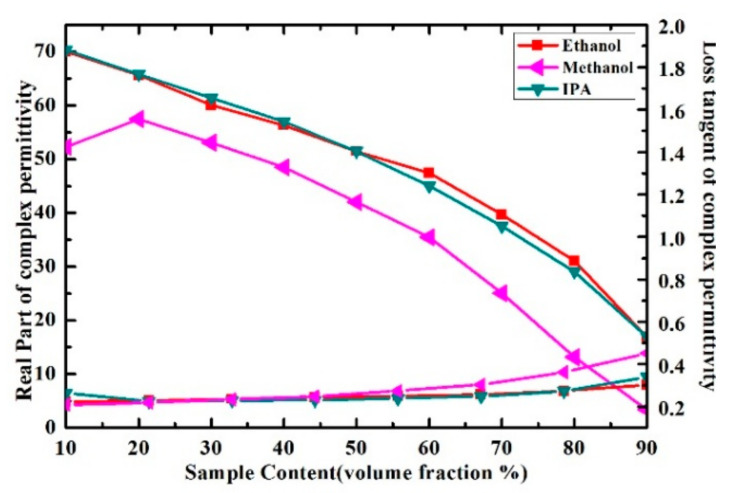
Measured results of real part and loss tangent of ethanol-water, methanol-water and IPA-water solution at the frequency of 5.05 GHz.

In order to verify the performance of proposed sensor for the characterization of the complex permittivity of liquid samples, the measurement results in [29] were compared with our measurement results. The measured real part of the complex permittivity of ethanol-water solution is well consistent with the literature results calculated by Debye model, as shown in Figure 13. While the loss tangent of the solution was inconsistent with the literature results when the volume fraction of ethanol changes from 30% to 70%, the disagreements between the measured and literature results of loss tangent may potentially arise from the measurement uncertainties and the simplified linear approximation of the measure model. Meanwhile, the measured results of real part and loss tangent of ethanol-water, methanol-water and IPA-water solution at the resonant frequency are shown in Figure 14. The real part of methanol solution can be clearly distinguished from the one of ethanol and IPA solution when we consider the same volume fraction. However, the real part of ethanol solution could not be distinguished from the one of IPA solution because the curves almost overlap, which means the proposed microwave sensor has room for improvement in the identification of liquid samples with such close complex permittivity.

At the same time, the performance of the proposed sensor is compared with other microwave sensor in Table 8. The item ‘Frequency (GHz)’, ‘Frequency shift (MHz)’ and ‘Volume (μL)’ represent the resonant frequency of sensor, the frequency changes of sensor when loaded sample and the volume of liquid sample, respectively. When we considered the volume of the sample, the sensitivity is defined as S = Δ*f*/Δ***ε***′_sam_/μL, where Δ*f* and Δ***ε***′_sam_ represent the frequency shift and the real part of sample as motioned above. The sensitivities of sensors in literature [33,34] and our proposed sensor are 12.73 MHz/μL, 9.09 MHz/μL and 97.46 MHz/μL, respectively. In addition, the proposed microwave sensor has advantages of non-contact, miniaturized, reusable and easily fabricated when compared with other non-MW sensor [35,36]. With these advantages, the microwave sensor provides a useful reference for the characterization of extremely small volume liquid samples in the field of biological and chemical.

## 5. Complex Permittivity Characterization of Inositol Solution

Inositol is an important intermediate product in the process of neurotransmitter transmission [38,39]. Therefore, it is of great significance for the complex permittivity characterization and concentration discrimination of inositol solution for human health care.

**Figure 15:**
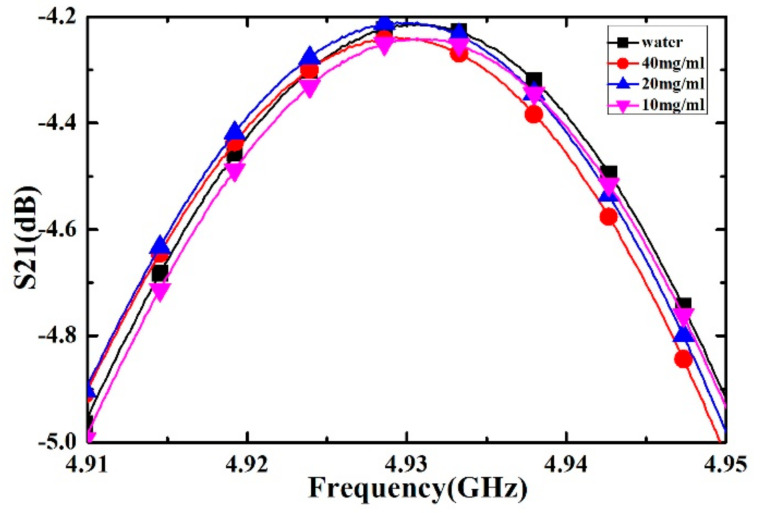
Measured transmission responses of different concentration of inositol solution.

Inositol powder with a purity of 99.6% was purchased from Shanghai Macklin Bio-chemical Co., Ltd. (Shanghai, China). The deionized water was purchased from Chengdu Kelong Chemical Co., Ltd. (Chengdu, China). Inositol solutions with concentrations of 10, 20 and 40 mg/mL are obtained by mixing the inositol powder and deionized water. For different concentrations of inositol solution and deionized water, the measured transmission response curves are shown in Figure 15, the corresponding frequency shifts and the Q factors of the transmission response are listed in Table 9. As the concentration of inositol solution increases, the frequency shift also increases. Therefore, we can qualitatively estimate the level of inositol concentration through the value of the frequency shift.

**Figure 16:**
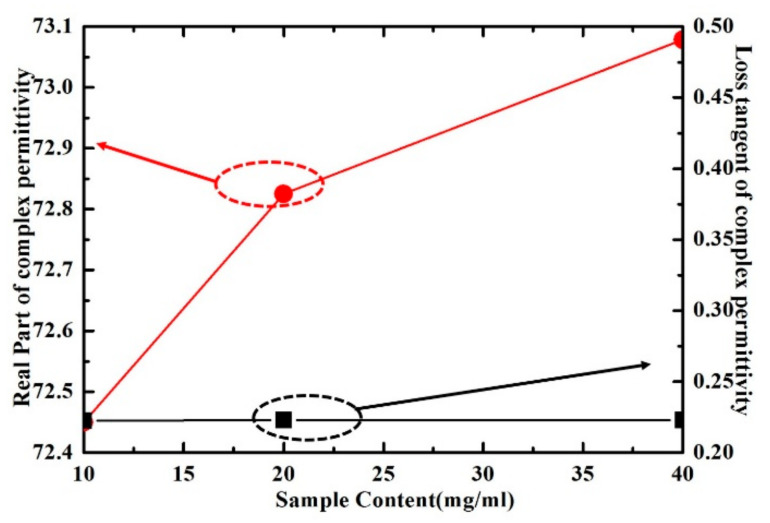
Measured results of real part and loss tangent of inositol solution with different concentration at the frequency of 5.05 GHz.

The real part and loss tangent of inositol solution with different concentration have been shown in Figure 16. As can be seen, the real part of the complex permittivity of inositol solution increases with the increase of concentration, while the loss tangent change is relatively small. These results show that the proposed sensor has the ability to distinguish the different concentrations of inositol solutions, which can be further improved on requirement.

## 6. Conclusions

In this paper, a sensor based on split ring resonator (SRR) used for the complex permittivity characterization of liquid samples has been proposed in the microwave frequencies range. Through the three-stage coupling transmission line, the quasi-TEM electromagnetic wave was coupled into the SRR. The SRR with two inner semi-circle was proposed to improve the Q factor of the sensor. The rectangular coupling area of structure was used to achieve the concentration of electric field. A quartz capillary channel was placed above the rectangular sensing area, and small change of the complex permittivity of liquid sample to be measured will cause a large change of resonance frequency and quality factor. The stop-flow technique has been used to inject liquid samples into the capillary channel for precise control of the sample volume. The complex permittivity of methanol, ethanol, isopropanol and deionized water at the resonance frequency have been obtained through the first order Debye model. Then complex permittivity of liquid sample can be calculated by the measured resonance frequency and Q factor. The complex permittivity of ethanol-water solution with different volume fractions have been characterized. Compared with other types of liquid and gas microwave sensor, our proposed sensor has a higher sensitivity reached 97.46 MHz/μL when considering the extremely small sample volume which is quite important in the field of biological and chemical sample characterization. The sensitivity of our proposed sensor is almost 11 times of the sensor mentioned in literature [34], which means our proposed sensor is with a higher sensitivity than the other sensors when considering the extremely small volume of the liquid samples. The complex permittivity characterization of inositol solutions with concentration of 10 mg/mL, 20 mg/mL and 40 mg/mL are achieved by using the calibrated sensor. It was proved that the proposed sensor has the ability to characterize complex permittivity and distinguish different concentration of liquid samples, while the sensitivity of the sensor can be further improved for such specific applications. At the same time, the proposed sensor based on SRR is miniaturized, low cost, label free and reusable. These advantages are promising for the extremely small liquid sample category analysis and complex permittivity characterization in chemical industry and healthcare field.

## Figures and Tables

**Table 1:** Parameter values of the proposed microwave sensor.

Parameters	Description	Values (mm)
w_1	The width of first coupling line	4.8
w_2	The width of second coupling line	1.4
w_3	The width of third coupling line	0.6
w_4	The width of coupling feedline	0.5
w	The width of SRR structure line	0.4
gap_1	The gap length between coupling line and SRR	0.6
gap_2	The gap length of coupling zone	0.3
gap_3	The gap length between SRR line	0.2
w_c	The width of coupling zone	0.4
l_c	The length of coupling zone	2
r_1	The radius of outer SRR line	3
r_2	The radius of inner SRR line	2.4

**Table 2:** The Debye model parameters of different liquid.

Sample	*ε*_∞_	*τ_D_* (ps)	Δ*ε_D_*
Ethanol	4.68	143.18	20.57
Methanol	6.58	52.63	26.66
Isopropanol	2.48	25.86	15.68
Deionized Water	4.55	7.37	72.56

**Table 3:** The resonant frequency and Q-factor of different liquid sample.

Sample	Frequency Shift (MHz)	Q-Factor
Empty	-	52.755
Ethanol	58.7	36.069
Methanol	89.6	37.940
Isopropanol	38.0	40.486
Deionized Water	116.9	49.644

**Table 4:** The resonant frequency and Q-factor of different concentrations of ethanol-water solution (from 1% to 9% with step of 1%).

Sample	Frequency Shift (MHz)	Q-Factor
Empty	-	52.755
1% Ethanol	116.73 ± 0.3	49.651 ± 0.421
2% Ethanol	116.65 ± 0.2	49.489 ± 0.254
3% Ethanol	116.64 ± 0.2	49.346 ± 0.213
4% Ethanol	116.56 ± 0.3	49.151 ± 0.416
5% Ethanol	116.52 ± 0.2	49.083 ± 0.189
6% Ethanol	116.39 ± 0.1	48.862 ± 0.233
7% Ethanol	116.19 ± 0.1	48.665 ± 0.205
8% Ethanol	116.07 ± 0.2	48.577 ± 0.241
9% Ethanol	115.91 ± 0.2	48.512 ± 0.322

**Table 5:** The resonant frequency and Q-factor of different concentrations of ethanol-water solution.

Sample	Frequency Shift (MHz)	Q-Factor
Empty	-	52.755
10% Ethanol	115.7	48.443
20% Ethanol	114.6	46.710
30% Ethanol	111.6	44.989
40% Ethanol	110.7	43.528
50% Ethanol	107.1	42.361
60% Ethanol	104.6	41.244
70% Ethanol	98.0	39.674
80% Ethanol	91.0	37.823
90% Ethanol	79.0	34.927

**Table 6:** The resonant frequency and Q-factor of different concentrations of methanol-water solution.

Sample	Frequency Shift (MHz)	Q-Factor
Empty	-	52.755
10% Methanol	106.1	51.283
20% Methanol	104.6	50.274
30% Methanol	105.2	48.891
40% Methanol	103.8	47.925
50% Methanol	103.6	46.283
60% Methanol	100.9	45.007
70% Methanol	101.3	42.753
80% Methanol	98.5	40.882
90% Methanol	94.7	38.159

**Table 7:** The resonant frequency and Q-factor of different concentrations of IPA-water solution.

Sample	Frequency Shift (MHz)	Q-Factor
Empty	-	52.755
10%IPA	98.4	50.840
20% IPA	96.7	48.959
30% IPA	96.6	46.747
40% IPA	94.6	45.076
50% IPA	92.1	42.611
60% IPA	89.5	40.147
70% IPA	84.0	36.685
80% IPA	74.8	33.762
90% IPA	65.2	32.071

**Table 8:** Comparison table of proposed sensor and other sensors.

Sample Form	Frequency (GHz)	Frequency Shift (MHz)	Volume (μL)	Sensitivity S (MHz*/ε′*_sam_*/*μL)
Ethanol liquid	4.60 [34]	70	1	12.73
Ethanol liquid	4.20 [33]	240	5	9.09
Ethanol Gas	4.20 [37]	150	Null	Null
Ethanol liquid	5.05 (our work)	58.7	0.13	97.46
Methanol liquid	5.05 (our work)	89.6	0.13	54.62
IPA liquid	5.05 (our work)	38.0	0.13	182.36

**Table 9:** The frequency shift and Q-factor of different concentration of inositol solution.

Sample	Frequency Shift (MHz)	Q-Factor
Empty	-	52.755
10 mg/mL Inositol	117.3	49.564
20 mg/mL Inositol	118.1	49.486
40 mg/mL Inositol	118.6	49.446

## Data Availability

Not applicable.
